# Bipolar disorder’s treatment and impulsivity

**DOI:** 10.1192/j.eurpsy.2021.532

**Published:** 2021-08-13

**Authors:** S. Ajmi, S. Najjar, J. Masmoudi

**Affiliations:** 1 Psychiatrie A, hospital University Hedi Chaker, sfax, Tunisia; 2 Psychiatry A, hedi chaker hospital, Sfax, Tunisia; 3 Psychiatrie “a” Department, Hedi Chaker Hospital University -Sfax - Tunisia, sfax, Tunisia

**Keywords:** Impulsivity, Mood stabilizer, antipsychotic, biopolar disorder

## Abstract

**Introduction:**

Impulsivity is not a classical psychiatric diagnosis like schizophrenia, depression, bipolar or borderline personality disorder. It is a symptom that could occur in almost all psychiatric disorders and in some neurological or systemic diseases.

**Objectives:**

In this study we examine the influence of bipolar disorder’s (BD) treatment on the impulsivity.

**Methods:**

We performed a cross sectional study on 30 patients diagnosed with BD and consulting at the Psychiatric department of HediChaker Hospital. Patients were euthymic during the time of the study confirmed by administration Young Mania Rating Scale (YMRS) and Montgomery Depression Rating Scale (MDRS). The socio-demographic data and treatment models were obtained. Impulsivity was evaluated using the Barratt Impulsiveness Scale (BIS-11).

**Results:**

The study sample consisted of 30 patients (10 men and 20 women). The mean age of the sample was 45.83 years (SD= 11. 63). Seventeen patients (56.7%) were married. Half of the participants were using an association of mood stabilizer (MS) and an antipsychotic (AP), 36.7% and 13.3% were receiving respectively only mood stabilizer or an antipsychotic. The mean BIS11 score was 75. 60 (SD=5.51) and 76.7% had a high level of impulsivity. No correlation was found between the level of impulsivity (BIS-11 scores) and using MS, AP or MS+AP (p=0.199; p=0.933; p=0.195).

**Conclusions:**

Further studies should be realized to identify pharmacological treatment of impulsivity among people with BD.

